# The implementation of an organised cervical screening programme in Poland: an analysis of the adherence to European guidelines

**DOI:** 10.1186/s12885-015-1242-9

**Published:** 2015-04-14

**Authors:** Andrzej Nowakowski, Marek Cybulski, Andrzej Śliwczyński, Arkadiusz Chil, Zbigniew Teter, Przemysław Seroczyński, Marc Arbyn, Ahti Anttila

**Affiliations:** 1 Department of Gynaecology and Oncologic Gynaecology, Military Institute of Medicine, ul. Szaserów 128, 04-141 Warsaw 44, Poland; 2 Department of Biochemistry and Molecular Biology, Medical University of Lublin, ul. Chodźki 1, 20-093 Lublin, Poland; 3 National Health Fund, Central Office, ul. Grójecka 186, 02-390 Warsaw, Poland; 4 Department of Oncologic Gynaecology, Regional Coordinating Office for Cervical and Breast Cancer Prevention Programmes, Świętokrzyskie Cancer Centre, ul. Artwińskiego 3, 25-734 Kielce, Poland; 5 ASSECO Poland, ul. Adama Branickiego 13, 02-972 Warsaw, Poland; 6 Unit of Cancer Epidemiology & Belgian Cancer Centre, Scientific Institute of Public Health, Juliette Wytsman Street, 14, B1050 Brussels, Belgium; 7 Finnish Cancer Registry, Unioninkatu 22, FI-00130 Helsinki, Finland

**Keywords:** Cervical cancer, Screening, Pap smear, Guidelines, Poland

## Abstract

**Background:**

Well-organised quality-controlled screening can substantially reduce the burden of cervical cancer (CC). European guidelines (EuG) for quality assurance in CC screening provide guidance on all aspects of an organised screening programme. Organised CC screening in Poland was introduced in 2007. The purpose of our study was to analyse: *(i)* adherence of the programme to EuG; *(ii)* programme process and performance indicators; *(iii)* impact of the programme on the incidence of and mortality from CC.

**Methods:**

Available data on the policy, structure and functioning of the Polish programme were compared with the major points of the EuG. Data on the process, and available performance indicators were drawn from the screening database and other National Health Fund (NHF) systems. Joinpoint regression was used to assess changes in CC incidence and mortality trends.

**Results:**

The Polish programme adheres partially to EuG in terms of policy and organisation. Only a limited set of performance indicators can be calculated due to screening database incompleteness or lack of linkage between existing databases. The screening database does not include opportunistic smears collected within NHF-reimbursed or private care. The organised programme coverage rate fluctuated from 21% to 27% between 2007-2013. In 2012 the coverage reached 35% after combining both organised and opportunistic smears reimbursed by the NHF. In 2012 the number of smears reimbursed by NHF was 60% higher in opportunistic than in organised screening with significant overlap. Data from the private sector are not recorded. Depending on years, 30-50% of women referred for colposcopy/biopsy because of abnormal Pap smears were managed within the programme. The age-standardised CC incidence and mortality dropped linearly between 1999 and 2011 without evidence of a period effect.

**Conclusions:**

The Polish organised cervical screening programme is only partially adherent to evidence-based EuG. Its implementation has not influenced the burden of CC in the country so far. Changes with special focus on increasing coverage, development of information systems and assessment of quality are required to increase programme adherence to EuG and to measure its effectiveness. Our findings may be useful to improve the Polish programme and those implemented or planned in other countries.

**Electronic supplementary material:**

The online version of this article (doi:10.1186/s12885-015-1242-9) contains supplementary material, which is available to authorized users.

## Background

There is a widely accepted consensus based on data from cohort [1], case-control [1,2] and modelling [3] studies that cytological screening followed by treatment of preinvasive cervical neoplasia results in a substantial reduction of the incidence of and mortality from cervical cancer (CC). The burden of CC is geographically heterogeneous throughout the world with highest incidence in low income countries lacking effective screening programmes [4]. Poland has a medium incidence of and mortality from CC among European countries with world-age standardised rates of 9.8/100 000 and 4.8/100 000 respectively in 2011 [5].

Well-organised high-quality cytological screening may reduce CC incidence substantially. The successive steps of successful screening include information and invitation of the eligible target population, performance of the screening test, follow-up and treatment of the screen-detected neoplasia [6-8].

CC prevention in Poland has been present for over 50 years now with opportunistic screening run since the 1950ties and the first active programme (for personal invitation) implemented locally in one district of Warsaw in 1976 [9]. However, active country-wide population-based screening was absent. In 2007, such a programme was set up with full registration of invitations, responses to invitations, results of Pap smears and other procedures in an internet-based electronic registry.

The purpose of this study was to analyse the adherence of the Polish programme to the European Guidelines (EuG) for Quality Assurance in Cervical Cancer Screening [7,8]. We also summarised the course and effects of the first seven years (over two screening rounds) of the programme in Poland.

## Methods

The use of data for this publication was approved by National Health Fund (NHF) authorities. The data for this study was drawn collectively from NHF databases and other available sources. All records were coded in such a way that identification of individuals was impossible.

### Policy, structure, parameters and performance indicators of the programme

Data on the policy, structure and functioning of the organised cervical screening programme were drawn from published documents of the Director of the NHF in Poland [10], previously published reports [11-17], as well as analysis of the internet-based electronic data base SIMP (abbreviated from Polish: System Informatyczny Monitorowania Profilaktyki; English: Informatic System for Monitoring of Prevention) [18].

Organised CC screening programme in Poland is a public healthcare intervention organised by the Ministry of Health and the NHF. In its current version it was started in 2006/2007. Every three years, all women aged 25-59 identified from the lists of General Practitioners’ (GP) practices (which cover ~95% of population of women) are sent a written invitation via ordinary mail to have a Pap smear taken. The invitations without a set time and date of appointment are sent after a 36-month interval from a previous normal smear performed in the programme. HPV- and HIV-infected women and those taking immunosuppressants are eligible to perform smears every year. No reminder letters are sent to non-responders. A gynaecological clinic in the neighbourhood of woman’s GP practice is suggested on the invitation, but a Pap smear may be taken by a gynaecologist or a certified midwife in any of the ambulatory clinics in the country which provide gynaecological and obstetrical care reimbursed by the NHF. Since 2014, certified family midwives are also eligible to collect smears at GPs’ practices. The smears are processed by cytotechnicians and pathologists in selected cytological laboratories. The laboratories evaluating smears within the programme must fulfill explicit criteria and are expected to perform internal quality control. This includes full reviewing of 10% of negative slides or rapid reviewing of all negative slides and control of all positive slides by a specialist pathologist. All labs undergo quality audits annually by external pathologists according to a protocol elaborated by the Central Coordinating Office. Underperforming labs are excluded from the programme. The smears are interpreted according to a modification of the Bethesda system [19] (See Additional file 1). Women with abnormal Pap smear results are supposed to undergo triage within the programme via: 1) repeated Pap smear (ASC-US - Atypical Squamous Cells of Undetermined Significance, LSIL – Low Grade Squamous Intraepithelial Lesion), or 2) colposcopy/colposcopically directed biopsy (ASC-US, LSIL, ASC-H - Atypical Squamous Cells - Cannot Exclude High Grade Squamous Intraepithelial Lesion, HSIL – High Grade Squamous Intraepithelial Lesion, AGC – Atypical Glandular Cells, SCC - Squamous Cell Carcinoma). If triage procedures are performed within the programme, the results are recorded in the SIMP. Human Papillomavirus (HPV) testing recommended for triage of ASC-US and LSIL [20] is not available in the programme but can be performed within NHF-reimbursed gynaecological services. Medical procedures in opportunistic screening and management of screen-positive women are reimbursed by the NHF within ambulatory and hospital gynaecological care outside the screening programme but results are not recorded in SIMP. Despite recommendations [21], there are no obligatory certification or quality requirements for cytological laboratories operating outside the screening programme.

Costs of Pap smear collection, evaluation as well as colposcopy and biopsies performed within the programme are fully covered by the NHF. Costs of administration, coordination of the programme as well as mailing of invitations are covered by the Polish Ministry of Health from the funds of the National Programme for Fight Against Cancer Act [22]. The screening programme is coordinated by the Ministry of Health, a Central and 16 Regional Coordinating Offices (RCO). The RCOs send invitations to women and are supposed to monitor, evaluate and perform quality control of the programme. They are also responsible for training of personnel involved in the program and for setting up interventions to increase the programme coverage. These interventions include, but are not limited to: local and countrywide media campaigns, cooperation with local authorities, governmental and non-governmental organisations, direct meetings with programme participants and healthcare providers, promotion of the programme via countrywide and local cultural and social events.

### Cancer registration

The National Cancer Registry (NCR) collects data via its 16 regional offices on cancer incidence, mortality, morbidity and survival in Poland [5]. Data on incidence come from electronic or traditional paper reports [5,23]. 87% of diagnoses were confirmed by pathology codes in 2011 [24]. Data on cancer mortality come from Central Statistical Office and are based on death certificates.

Since adherence of the programme to some points of the EuG [7,8] cannot be measured in a quantitative manner, a descriptive comparison of the valid recommendations, legislative acts and real-life clinical practice to the major points of the EuG is given. The adherence of the programme was qualified as full, partial or absent according to the level of agreement between the EuG and the Polish programme. Data on the available parameters and outcome measures of the CC screening programme in Poland were drawn from SIMP and other NHF electronic databases. We analysed which of the performance parameters in CC screening recommended by the EuG [7] can be calculated for Poland.

### Burden of invasive cervical cancer

World age-standardised incidence and mortality rates of CC in Poland were extracted from the NCR database in Poland [5]. Rates were aggregated by calendar year and 10-year age groups (except for the oldest women categorised as ≥ 80 years). Joinpoint regression was used to analyse time trends (Joinpoint Regression Software) [25]. Joinpoint regression identifies periods with distinct linear slopes that can be separated by joinpoints, where the slope of the trends changes significantly [26]. For each linear segment, the annual percent change (APC) and corresponding 95% confidence intervals (95% CI) were calculated. Analysis was performed for a 13-year period from 1999 to 2011 which encompassed years of implementation of the screening programme (2006/2007).

## Results

### Policy and structure of the programme

The adherence of the most important constituents of the Polish programme to EuG is summarised in Table 1. The Polish programme is fully adherent to the EuG in terms of: type of screening test; interval between tests with normal results; and the age to start testing (Table 1). Partial adherence is noted for: the type of screening and screening policy; the age to stop testing; the issue of older women who have never attended screening; discouragement for opportunistic screening; information system; publication of programme indicators and new screening technologies (Table 1).

**Table 1:** **Adherence of the organised cervical screening programme in Poland to European Guidelines for Quality Assurance in Cervical Cancer Screening** [7,8] **– screening policy, organisation, monitoring, evaluation and new screening technologies**

Point of the guideline	Recommendation of the guidelines	Adherence of the Polish programme to the guideline	
		Legal regulations, guidelines and protocols	Implementation and clinical practice
Screening type	Population-based public healthcare programme, with identification and personal invitation of each woman in the eligible target population	Partial adherence	
		Full adherence.	Postage of invitations is not regular^a^.
Screening policy	Selection of screening test systems, determining target age group and interval between normal test results, establishing follow-up and treatment strategies for screen positive women	Partial adherence	
		Treatment strategies are not included in organised screening policy.	Large part of triage of abnormal Pap smears is performed outside the programme^b^.
Screening test	Cytology	Full adherence	
Interval between tests with normal results	3-5 years	Full adherence	
Age to start testing	20-30 years of age	Full adherence	
Age to stop testing	60-65 years of age. Stopping screening in older women who have had three or more consecutive recent normal cytology results.	Partial adherence	
		No system of stopping organised screening in older women with previous normal smears has been elaborated.	Opportunistic screening in older women is reimbursed and performed regardless of screening history.
Issue of older women who have never attended screening	Special attention should be paid to older women who have never attended screening as they are at increased risk for CC	Partial adherence	
		No systemic solutions have been undertaken to reach women older than 59 who have never attended screening despite unfavourable epidemiological data^c^. Women older than 59 are not allowed to undergo organised testing regardless of screening history.	Coordinating Offices and the Ministry of Health undertake multiple actions to increase coverage of the programme and to reach non-attenders among women at the screening age 25-59.
Opportunistic screening	Opportunistic screening should be discouraged. It leads to “overscreening” of selected populations and “underscreening” of groups with less socioeconomic status.	Partial adherence	
		Educational campaigns led by Coordinating Offices have been introduced to discourage opportunistic screening but it is reimbursed and recommended in pregnancy [43].	Private-based opportunistic screening is popular but its extend and outcomes has never been precisely assessed. There are non-governmental initiatives encouraging opportunistic screening at one year intervals in young age groups [44].
Information system	Implementation of the information system for managing the screening programme; computing the indicators of attendance, compliance, quality and impact and providing feedback.	Partial adherence	
		The implemented system (SIMP) enables computation of selected indicators from organised screening only.	Only partial data on screening outcomes have been computed and analysed [11-17].
Linkage between information systems and databases	An appropriate legal framework is required for registration of individual data and linkage between population databases, screening files, cancer and mortality registers.	Partial adherence	
		A screening registry (SIMP) is implemented but not fully integrated with other existing systems and some registries are lacking^d^.	There is routine input of data into several systems, but they are not integrated.
Publication of programme indicators	Indicators of screening programme extension and quality need to be published regularly.	Partial adherence	
		Data available in SIMP are insufficient to generate some of the crucial indicators for publication.	Only selected indicators of the programme were published regularly by the Central Coordinating Office [11-17].
New screening technologies	Before routine implementation of new screening technologies phased piloting or even controlled randomised implementation should be executed for its evaluation under real-life conditions.	Partial adherence	
		Randomisation of screening policies is technically feasible. Pilot programme of primary HPV testing is on the way in two regions.	Comprehensive evaluation of pilot HPV testing will be hampered by partial availability of data on the outcomes in SIMP.

Footnotes: ^a^The postage is infrequent during the first 3-6 months of each year because of the late signature of contracts between the Ministry of Health and the Regional Coordinating Offices. ^b^Pricing of the triage (colposcopy/biopsy) is lower in the programme than outside within NHF-reimbursed procedures. ^c^Crude incidence rates of CC in Poland still remain high to the age of 84 [5].^d^Data from SIMP are partially linked with treatment databases of the National Health Fund but not with National Cancer Registry. SIMP enables reporting of data to the NCR but not obtaining data from the NCR e.g. to identify false negative Pap smear or colposcopy/histology results and interval cancers. Histology results of false negative cytology cases are not available in the SIMP; only partial data on type of treatment is available. The SIMP is linked to mortality registry but causes of deaths are not available in SIMP. There is no registry of Pap smears or cervical histology results obtained outside the programme.

The adherence of organised screening in Poland to EuG for cytology laboratories, histopathology, management of screen-positive women is presented in Table 2. The Polish programme is fully adherent to EuG in the aspect of: collection, preparation, handling, staining, screening of samples and reporting of the results; and partially adherent regarding: grading of cytological abnormalities; histopathology as the gold standard and its terminology; availability of cytological results for pathologists, accuracy of histological diagnosis, correlation with cytological results; and management of screen-positive women (Table 2) [27-32]. No major points in the Polish screening programme have been identified to be completely non-adherent to EuG.

**Table 2:** **Adherence of the organised cervical screening programme in Poland to European Guidelines for Quality Assurance in Cervical Cancer Screening** [7,8,27-32] **– guidelines for cytology laboratories, histopathology, management of screen-positive women**

Point of the guideline	Recommendation of the guidelines	Adherence of the polish programme to the guideline	
		Legal regulations, guidelines and protocols	Implementation and clinical practice
Collection, preparation, handling, staining, screening of samples and reporting of the results	Guidelines must be followed to assure adequate collection and preparation of the samples. The quality of the cytology laboratory depends on adequate handling, staining, screening of slides and reporting of results.	Full adherence	
Grading of cytological abnormalities	Uniform grading of cellular abnormalities is an essential condition for registration and comparisons. Laboratories should apply only a nationally agreed terminology for cytology which is translatable into the Bethesda Reporting System	Partial adherence	
		The grading system is not fully compatible with the Bethesda 2001 terminology and requires modification (see Additional file 1)	Full adherence to established grading system
Histopathology as the gold standard and its terminology	Histopathology provides the final diagnosis for treatment, is the gold standard for quality control of cytology and colposcopy and is the source of data for cancer registry. Histopathology standards should be monitored and are on the basis of CIN or other internationally agreed-upon terminology.	Partial adherence	
		There is no electronic database of cervical histology results obtained outside the programme. No systematic quality control for histopathology is implemented into the screening programme. There is no automatic or obligatory reporting of histology from the labs to cancer registry.	Only partial data on histopathology of invasive cancers are collected in NCR.
Availability of cytological results for pathologists, accuracy of histological diagnosis, correlation with cytological results.	Histopathologists should be aware and familiar with, the nature of cytological changes that may be relevant to their reports. The accuracy of histopathological diagnosis depends on adequate samples, obtained by colposcopically directed biopsies (with endocervical curettage when necessary) or excision of the transformation zone or conisation, macroscopic description, processing, microscopic interpretation and quality management correlating cytological and histological diagnosis.	Partial adherence	
		There is no central histopathology database and therefore cervical histology results obtained outside the programme are not readily available for analyses and cyto-histological correlations.	Tissue material from biopsies is often assessed at different laboratories than the ones assessing the cytological slides. The availability of data on cytological abnormalities to the pathologists is partial^a^. Only single local reports exist [51] on local correlations between cytology and histopathology.
Management of screen-positive women	A women with a high-grade cytological lesion, a repeated low-grade lesion or an equivocal cytology results and a positive HPV test should be referred for colposcopy. Guidelines are provided for the management of ASC-US and HSIL. For LSIL repeat cytology or colposcopy are acceptable options and HPV testing in older women can be considered. Quality assurance and collection of data on patient management and follow-up are important in women with abnormal cytology.	Partial adherence	
		HPV testing is not reimbursed within the programme for triage of abnormal Pap results. Only partial data on management of women with abnormal smears are available in SIMP.	Repeat cytology and other triage procedures are commonly performed outside the programme and their results are not registered. Data on triage, management and follow-up are not evaluated and not analysed on regular bases.

Footnotes: ^a^It is not automatic and depends on the quality of information from the clinician on the referral letter to the pathologist.

### Parameters and performance indicators of the programme

Selected available parameters of the organised and reimbursed opportunistic screening are presented in Table 3. The data collected in SIMP and other systems enable calculation of the following performance indicators [7]: programme extension; coverage of the target population by invitation; coverage of the target population by smear tests; compliance to invitation; distribution of screened women by the results of cytology; referral rate for repeat cytology; compliance to referral for repeat cytology; referral rate to colposcopy; positive predictive value of referral to colposcopy; compliance to referral to colposcopy.

**Table 3:** **Selected available parameters on cervical cancer screening in Poland after implementation of organised screening programme (2007-2013)**

Parameter/year	2007	2008	2009	2010	2011	2012	2013
Population eligible for screening – 1/3 of the population of women aged 25-59 years	3 227 918	3 252 888	3 274 036	3 289 805	3 293 187	3 293 976	3 290 725
Number of invitations sent	6 027 714	2 682 051	1 595 319	3 202 927	3 357 114	3 413 678	3 220 582
Coverage of population by invitations	186.7%	82.5%	48.7%	97.4%	101.9%	103.6%	97.9%
Compliance to invitations*	11.2%	12.8%	13.1%	11.5%	11.2%	10.7%	9.9%
Number of women screened within the programme	686 380	794 486	876 646	797 442	804 847	765 301	696 894
Coverage rate of organised screening	21.3%	24.4%	26.8%	24.2%	24.4%	23.2%	21.2%
Total number of Pap smears collected within NHF outside the screening programme	NA	NA	NA	NA	NA	1 288 358	NA
Total number of Pap smears collected in women aged 25-59 years within NHF outside the screening programme	NA	NA	NA	NA	NA	807 129	NA
Number of women screened outside the programme within NHF opportunistic screening and not screened within the programme within the current 3-year interval	NA	NA	NA	NA	NA	411 216	NA
Combined coverage of organised and opportunistic screening within NHF	NA	NA	NA	NA	NA	35.7%	NA
Number of women referred for colposcopy/biopsy within the programme	4 917	6 149	9 158	9 216	9 850	9 879	10 496
% of women referred for colposcopy/biopsy who underwent colposcopy/biopsy within the programme	41.1%	50.5%	46.8%	34.2%	31.7%	31.2%	29.7%

Legend: *Data with a 6-month cut-off date generated on 30^th^ of June of each following year. Data on women screened within opportunistic screening provided within private gynaecological care and private insurance plans are unknown since there is no central registry of opportunistic smears. NA - data not available.

The following indicators cannot be automatically calculated based on the available systems: smear consumption; incidence of invasive cancer in unscreened or underscreened women in a given interval; test specificity; detection rate by histological diagnosis; treatment of high-grade intraepithelial lesions; proportion of women hysterectomised on screen-detected intraepithelial lesions; proportion of women treated for Cervical Intraepithelial Neoplasia grade 1 (CIN1); incidence of invasive cancer after normal and after abnormal cytology; proportion of women with cytology negative for squamous intraepithelial lesions, 6 months after treatment.

At this stage, smear consumption can be calculated by merging data from SIMP with data on opportunistic NHF-reimbursed Pap smears but not automatically on regular basis. Pap smear consumption in private care and private insurance plans is not recorded. Indicators such as incidence of cervical cancer in unscreened or underscreened women in a given interval, cancer incidence after normal or after abnormal cytology, treatment of high-grade intraepithelial lesions, proportion of women hysterectomised on screen-detected lesions theoretically can be calculated based on data from NHF treatment databases containing International Classification of Diseases version 10 (ICD10) codes and types of procedures with International Classification System for Surgical, Diagnostic and Therapeutic Procedures (ICD-9-CM) codes. However at present it would require special searches in the electronic systems and the quality of these data is undetermined since they contain no histological reports. NCR database and SIMP have not been connected electronically so far and computation of certain indicators is therefore still impossible at present.

### Burden of invasive cervical cancer

**Figure 1:**
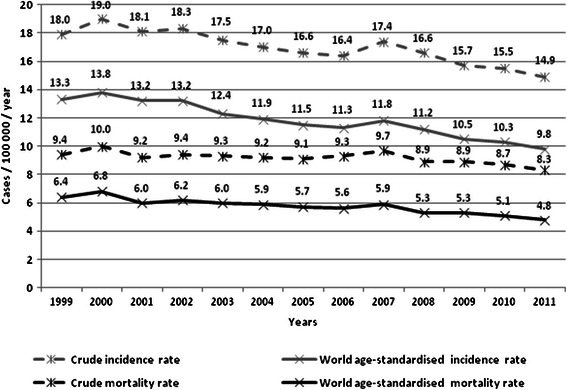
Cervical cancer burden in Poland between 1999 and 2011.

**Figure 2:**
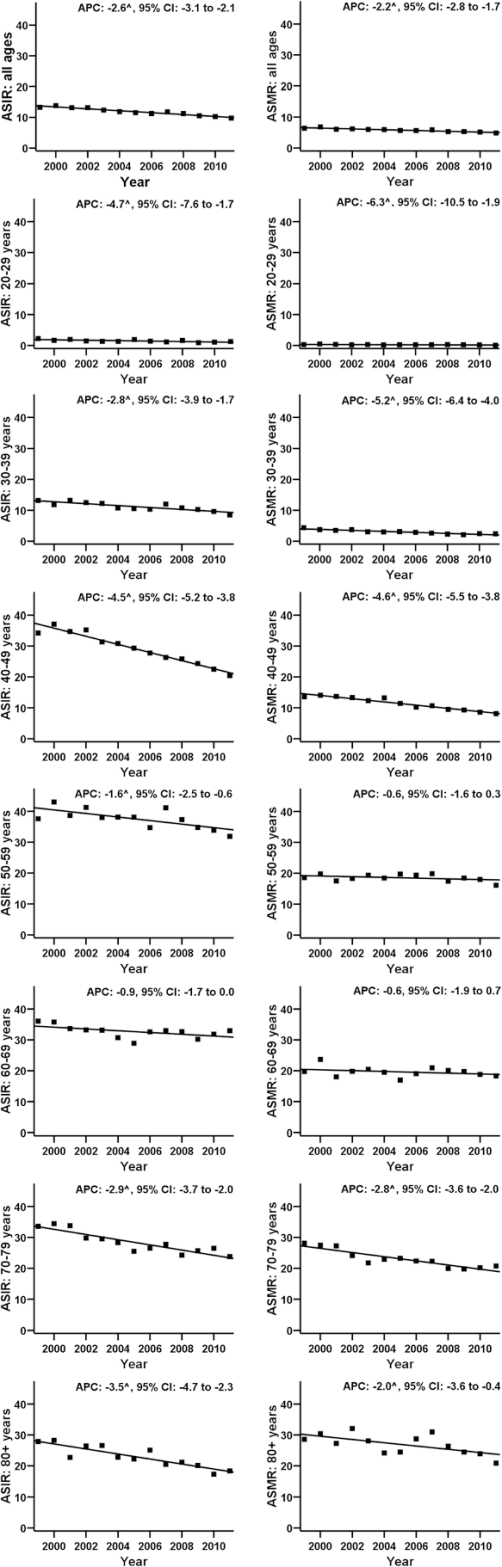
World age-standardised rates of incidence of and mortality from cervical cancer in Poland (1999-2011). Figure legend: ASIR - Age-Standardised Incidence Rate; ASMR - Age-Standardised Mortality Rate; ^ - The Annual Percent Change (APC) is significantly different from zero; 95% CI - 95% confidence interval.

Both age-standardised CC incidence and mortality rates have been decreasing steadily for the last decade in Poland (Figure 1). Figure 2 shows the age-standardised incidence rates (ASIR), and mortality rates (ASMR), as well as the annual percentage change (APC) with 95% CIs for CC in Poland (1999-2011). The declines in both standardised rates decreased significantly in the period 1999-2011 with the APC for incidence: -2.6, 95% CI: -3.1 to - 2.1 and the APC for mortality: -2.2, 95% CIN: -2.8 to -1.7 (Figure 2). The decreases in incidence were significant in all 10-year age groups apart from women aged 60-69 years (Figure 2). The age-specific mortality rates dropped significantly in all age groups apart from women aged 50-59 and 60-69 years (Figure 2). The linear slopes were constant in all age groups and no significant trend changes were identified over the analysed period (1999-2011) encompassing implementation of the programme (2006/2007) (Figure 2).

## Discussion

The implementation of organised cervical screening programmes in the member states of the European Union was recommended by the European Council in 2003, in agreement with EuG [7]. After an initial phase between 2004-2006, an organised programme was rolled out in its current state in 2007 in Poland. The trend of the burden of CC may be indicative of the impact of the enrolment of preventive programmes. Our initial analysis of the age-specific trends in CC incidence and mortality indicates no evidence of a significant period effect attributable to the introduction of organised screening (Figure 2). We consider these results with caution being aware that data from a simple age-period trend analysis might not be a sufficient proof of programme ineffectiveness. Additional more complex analyses utilising linkage between screening and cancer registries and incorporating data on changes in exposure to risk factors, changes in coverage and quality of non-monitored screening will be required to assess the impact of the implemented screening on the CC burden in Poland over a longer time perspective.

Our analysis reveals that organised CC screening in Poland is only partially adherent to EuG and there are a number of issues which require further insight and actions. The coverage and quality of screening seem to be most important. No registration of Pap smears collected outside the organised screening hampers comprehensive assessment of the coverage. In 2012 the coverage rate of the NHF-reimbursed (organised and opportunistic) screening reached 36% (Table 3). However according to survey-based data from Central Statistical Office in Poland, 86% of women aged 20-69 at least once in their lifetime have undergone pap testing and 73% of women at this age underwent a pap test within the preceding three-year interval [33]. Although, these questionnaire-based results probably overestimate the real coverage [34], they indicate that many women participate in the private-based opportunistic screening.

Organized screening is more effective and more cost-effective [7], however in many countries opportunistic screening is the only or dominating way of secondary prevention [35,36]. In Poland a direct shift from opportunistic to organised screening may be difficult to achieve in a short time due to habits of women and healthcare providers. Therefore integration of both screening modalities should be considered, as in other countries [35]. Analysis of systems integrating both types of screening in countries with longer experience such as Finland [37], the Netherlands [38], Denmark [39] and France [40] should be performed to select a model which fits best into Polish conditions. Nevertheless, more research on the reasons for non-attendance to the organised programme is required in order to find targeted solutions. Very recent findings from Finland indicate that carefully managed invitation/reminder letters with scheduled appointments and self-sampling options offered to non-attending women can increase organised programme effectiveness [41]. On the other hand, Belgian experience shows that sending invitations hardly influences screening coverage in a country with a long tradition of opportunistic screening [42]. Appropriate trials run in the real-life conditions are required to demonstrate the effectiveness of actions planned in Poland to increase organised screening coverage before costly implementation.

EuG propose discouragement of yearly opportunistic screening but opportunistic smears are still reimbursed by NHF and are recommended in pregnancy by the Ministry of Health [43] and by other actively operating non-governmental organisations in Poland [44]. No reimbursement for opportunistic smears could reduce overscreening in some cohorts, promote screening at recommended intervals and drive shifts towards organised screening [42]. However, stopping reimbursement of opportunistic screening might be controversial both for women and most of gynaecologists in Poland in the initial years. Nonetheless, it may induce better adherence of the real-life practice to the EuG and finally result in increased programme effectiveness. In Poland smears outside the screening programme are used and reimbursed for many clinical indications such as: triage of previously abnormal cytology, follow-up after treatment of CIN and cancer. A reimbursement protocol would have to be developed to reimburse smears for the above mentioned indications but not for screening beyond the recommended target age group and screening interval.

As explained in results, assessment of screening performance as recommended in EuG is currently impossible in Poland since several activities take place outside the organised programme, are not recorded and/or are inaccessible by lack of effective data linkage. Participation in the screening programme requires signing a consent for collection, storage and processing of womens’ personal data. However an additional legal framework is needed for data collection, storage, processing, including linkage of screening, follow-up and cancer register allowing to run and to evaluate the programme [7].

Although cytological labs in the programme undergo external quality audits every year, their results have not been published and assessed to reach conclusive points for programme improvements. Some of the cytological labs working outside the programme in the opportunistic screening are not monitored and their quality is unknown. Data on mortality audits and interval cancers have been published for some countries [45], but no such attempts have been made for Poland yet. Quality of colposcopy, and histopathology should be assured as well [46].

Consideration of new evidence regarding better performance of primary HPV-based compared to cytology-based screening is important for the country [47]. A pilot study on the use of HPV testing as a primary screening co-test has started in two regions. However results of HPV testing are not registered in SIMP which will hamper performance evaluation.

Implementation of a cancer screening programme can be divided into seven phases: (1) pre- planning, (2) planning, (3) feasibility testing, (4) piloting or trial implementation, (5) scaling up from pilot to service, (6) running, and (7) sustaining the full-scale programme [48]. For each phase, a substantial number of specified conditions have to be met. The Polish experience shows that some of the major points such as feasibility testing and pilot studies were omitted and some important detailed conditions such as the incorporation of an IT-system linking registries and a comprehensive system covering all steps in the screening process, development of a quality assurance plan and publication of its results have not been fully implemented thus far.

There may be other factors than the performance of organised screening, which may explain the higher rates of mortality from CC in Poland than in most of Western European countries [49]. The exposure to risk factors associated with cervical cancer as well as the effectiveness of diagnosis and treatment of women with invasive or preinvasive disease are among them and require further insight [50].

## Conclusions

Polish organised cervical screening programme is only partially adherent to evidence based EuG. Its implementation has had no impact on the burden of CC in the country so far. Comprehensive research of non-attendance to organised screening and targeted actions are required to increase its coverage. Development of information systems to obtain data on opportunistic smears and histology reports, and linkage with cancer registry data is required to increase programme adherence to EuG and to measure its effectiveness. Our findings may be useful to improve the Polish programme and those implemented or planned in other countries.

## Additional file

Additional file 1:
**The system used for classification of Pap smear results in organised cervical cancer screening programme in Poland – current version in use from 1st April 2014.**

## Abbreviations

CC: Cervical cancer
EuG: European guidelines
NHF: National Health Fund
SIMP: System Informatyczny Monitorowania Profilaktyki (Polish), Informatic System for Monitoring of Prevention (English)
GP: General Practitioner
ASC-US: Atypical squamous cells of undetermined significance
LSIL: Low grade squamous intraepithelial lesion
ASC-H: Atypical squamous cells, cannot exclude high grade squamous intraepithelial lesion
HSIL: High grade squamous intraepithelial lesion
AGC: Atypical glandular cells
SCC: Squamous cell carcinoma
HPV: Human papilloma virus
RCO: Regional Coordinating Office
NCR: National Cancer Registry
APC: Annual percent change
CI: Confidence interval
CIN1: Cervical intraepithelial neoplasia grade 1
ICD10: International Classification of Diseases version 10
ICD-9-CM: International Classification System for Surgical, Diagnostic and Therapeutic Procedures
ASIR: Age-standardised incidence rates
ASMR: Age-standardised mortality rates
IT-system: Information technology system
