# Supplementary material for: The implementation of an organised cervical screening programme in Poland: an analysis of the adherence to European guidelines
Source: BMC Cancer. 2015 Apr 14;15:279. doi: 10.1186/s12885-015-1242-9 (PMC4417537; doi:10.1186/s12885-015-1242-9)
Supplement: Additional file 1: — The system used for classification of Pap smear results in organised cervical cancer screening programme in Poland – current version in use from 1st April 2014. [file 12885_2015_1242_MOESM1_ESM.docx]

Additional file 1

The system used for classification of Pap smear results in organised cervical cancer screening programme in Poland – current version in use from 1st April 2014.

| Polish | | | English | | |
| --- | --- | --- | --- | --- | --- |
| A. Ocena techniczna rozmazu | Rozmaz odpowiedni do oceny |  | A. Technical assessment of the smear | Pap smear adequate for evaluation |  |
|  | Nie nadaje się do oceny cytologicznej | Rozmaz zbyt ubogokomórkowy |  | Pap smear inadequate for evaluation | Pap smear with too small cellularity |
|  |  | Rozmaz wysuszony przed utrwaleniem |  |  | Pap smear dried out before fixation |
|  |  | Rozmaz źle utrwalony |  |  | Pap smear inadequately fixed |
|  |  | Rozmaz nieczytelny z powodu licznych komórek zapalnych |  |  | Pap smear unreadable due to numerous inflammatory cells |
|  |  | Rozmaz nieczytelny z powodu licznych erytrocytów |  |  | Pap smear unreadable due to numerous erythrocytes |
|  |  | Rozmaz nieczytelny z innych powodów |  |  | Pap smear unreadable due to other reasons |
| B. Ocena ogólna rozmazu | Nie stwierdzono zmian śródnabłonkowych i procesu złośliwego | | B. General assessment of the smear | No intraepithelial lesions and malignancy found | |
|  | Stwierdzono nieprawidłowe komórki nabłonkowe | |  | Abnormal epithelial cells present | |
| C. Interpretacja/  wynik  (opis) | Stwierdzono czynniki infekcyjne – mikroorganizmy | Infekcja Trichomonas vaginalis | C. Interpretation/  result  (descritpion) | Infectious agents – microorganisms  found in the Pap smear | Infection with Trichomonas vaginalis |
|  |  | Infekcja grzybicza odpowiadająca Candida sp. |  |  | Fungal infection corresponding to Candida sp. |
|  |  | Infekcja o cechach bacterial vaginosis |  |  | Infection with features of bacterial vaginosis |
|  |  | Niespecyficzna infekcja bakteryjna |  |  | Unspecified bacterial infection |
|  |  | Infekcja bakteryjna o cechach Actinomyces |  |  | Bacterial infection with features of Actinomyces |
|  |  | Zmiany w komórkach odpowiadające infekcji wirusem HSV |  |  | Cellular changes corresponding to HSV infection |
|  |  | Zmiany flory bakteryjnej |  |  | Shifts in bacterial flora |
|  |  | Zmiany komórkowe odpowiadające Chlamydia T. |  |  | Cellular changes corresponding to Chlamydia T. |
| C. Interpretacja/  wynik  (opis) | Stwierdzono inne zmiany nienowotworowe – odczynowe i naprawcze | Zmiany odczynowe związane z zapaleniem (łącznie z typową regeneracją) | C. Interpretation/  result  (descritpion) | Other non-neoplastic changes - reactive and regenerative found | Reactive changes associated with inflammation (including typical regeneration) |
|  |  | Zmiany odczynowe związane z atrofią |  |  | Reactive changes associated with atrophy |
|  |  | Zmiany odczynowe związane z atrofią i zapaleniem |  |  | Reactive changes associated with atrophy and inflammation |
|  |  | Zmiany odczynowe związane z napromieniowaniem – radioterapią |  |  | Reactive changes associated with irradiation – radiotherapy |
|  |  | Zmiany odczynowe związane z odpowiedzią na ciało obce (IUD) |  |  | Reactive changes associated with response to foreign body (IUD) |
|  |  | Obecność komórek gruczołowych u kobiet po wycięciu macicy |  |  | Presence of glandular cells in women after hysterectomy |
|  |  | Obecność komórek gruczołowych u kobiet po 40 roku życia |  |  | Presence of glandular cells in women after 40 years of age |
|  | Nieprawidłowości komórek nabłonkowych | |  | Abnormal epithelial cells found | |
|  | Stwierdzono nieprawidłowe komórki nabłonka płaskiego | Nieprawidłowe komórki nabłonka płaskiego nieokreślonego znaczenia (ASC-US) |  | Presence of abnormal squamous cells | Abnormal squamous cells of undetermined significance (ASC-US) |
|  |  | Nieprawidłowe komórki nabłonka płaskiego nieokreślonego znaczenia, nie można wykluczyć HSIL (ASC-H) |  |  | Abnormal squamous cells cannot exclude HSIL (ASC-H) |
|  |  | LSIL - zmiany śródnabłonkowe stopnia niskiego obejmujące koilocytozę (HVP) i możliwość CIN1 (dysplazji małego stopnia) |  |  | LSIL – low grade intraepithelial lesion including koilocytosis (HPV) and possibility of CIN1 (low grade dysplasia) |
| C.Interpretacja/  wynik  (opis) | Nieprawidłowości komórek nabłonkowych | | C.Interpretation/  result  (descritpion) | Abnormal epithelial cells found | |
|  | Stwierdzono nieprawidłowe  komórki nabłonka płaskiego | HSIL - zmiany śródnabłonkowe stopnia wysokiego mogące odpowiadać CIN2, CIN3/CIS (dysplazji średniego i dużego stopnia)/ |  | Presence of abnormal squamous cells | HSIL – high grade intraepithelial lesion which might correspond to CIN2,CIN3/CIS (medium and high grade dysplasia) |
|  |  | Rak płaskonabłonkowy |  |  | Squamous cell carcinoma |
|  | Stwierdzono nieprawidłowe  komórki nabłonka gruczołowego | Nieprawidłowe komórki nabłonka gruczołowego (AGC): kanału szyjki (endocerwikalne) |  | Abnormal glandular cells found | Abnormal glandular cells (AGC): endocervical |
|  |  | Nieprawidłowe komórki nabłonka gruczołowego (AGC): endometrioidalne |  |  | Abnormal glandular cells (AGC): endometrial |
|  |  | Nieprawidłowe komórki nabłonka gruczołowego (AGC): nieokreślone |  |  | Abnormal glandular cells (AGC): undetermined |
|  |  | Nieprawidłowe komórki gruczołowe, prawdopodobnie nowotworowe: endocerwikalne |  |  | Abnormal glandular cells, favor neoplastic endocervical |
|  |  | Nieprawidłowe komórki gruczołowe, prawdopodobnie nowotworowe: endometrioidalne |  |  | Abnormal glandular cells, favor neoplastic endometrial |
|  |  | Gruczolakorak - adenocarcinoma endocervicale in situ (ACIS) |  |  | Endocervical adenocarcinoma in situ (ACIS) |
|  |  | Gruczolakorak - adenocarcinoma kanału szyjki |  |  | Adenocarcinoma of the cervical canal |
|  |  | Gruczolakorak - adenocarcinoma endometrioidale |  |  | Adenocarcinoma of the endometrium |
|  |  | Gruczolakorak - adenocarcinoma endometrioidale |  |  | Adenocarcinoma endometriale – extrauterine |
|  |  | Gruczolakorak - adenocarcinoma inny |  |  | Other adenocarcinoma |
